# Linezolid resistance in multidrug-resistant mycobacterium tuberculosis: A systematic review and meta-analysis

**DOI:** 10.3389/fphar.2022.955050

**Published:** 2022-08-30

**Authors:** Taher Azimi, Saeed Khoshnood, Arezoo Asadi, Mohsen Heidary, Hassan Mahmoudi, Vahab Hassan Kaviar, Masoume Hallajzadeh, Mohammad Javad Nasiri

**Affiliations:** ^1^ Department of Bacteriology and Virology, School of Medicine, Shiraz University of Medical Sciences, Shiraz, Iran; ^2^ Clinical Microbiology Research Center, Ilam University of Medical Sciences, Ilam, Iran; ^3^ Student Research Committee, Ilam University of Medical Sciences, Ilam, Iran; ^4^ Endocrine Research Center, Institute of Endocrinology and Metabolism, Iran University of Medical Sciences (IUMS), Tehran, Iran; ^5^ Department of Laboratory Sciences, School of Paramedical Sciences, Sabzevar University of Medical Sciences, Sabzevar, Iran; ^6^ Cellular and Molecular Research Center, Sabzevar University of Medical Sciences, Sabzevar, Iran; ^7^ Department of Microbiology, Faculty of Medicine, Hamadan University of Medical Sciences, Hamadan, Iran; ^8^ Nahavand School of Allied Medical Sciences, Hamadan University of Medical Sciences, Hamadan, Iran; ^9^ Microbial Biotechnology Research Center, Iran University of Medical Sciences, Tehran, Iran; ^10^ Department of Microbiology, School of Medicine, Iran University of Medical Sciences, Tehran, Iran; ^11^ Department of Microbiology, School of Medicine, Shahid Beheshti University of Medical Sciences, Tehran, Iran

**Keywords:** tuberculosis, meta-analysis, linezolid, TB, MDR-TB, resistance

## Abstract

**Introduction:** Linezolid (LNZ) is an effective antibiotic to treat patients with multidrug-resistant tuberculosis (MDR-TB) treatment failure. *M. tuberculosis* strains resistant to isoniazid and rifampin are defined as MDR-TB. In recent years, resistance to LNZ among MDR-TB cases has been reported in several different countries. In this study, we performed a systematic review and meta-analysis to investigate the prevalence of LNZ resistance among MDR-TB isolates.

**Methods:** The databases of Embase, PubMed/Medline, and Web of Science were searched systematically from January 2000 to April 2021. Statistical analyses were performed by using Comprehensive Meta-Analysis software. Heterogeneity was reported by using the t-squared statistic and Q-statistic. Begg’s rank correlation in combination with the funnel plot were used to evaluate any possible publication bias.

**Results:** In total, 25 studies were selected for meta-analysis from 14 different countries; the majority was from China (n = 5) and Turkey (n = 4). Moreover, 7,366 patients were infected with MDR *M. tuberculosis*. Among the study population, 98 patients were co-infected with HIV, and 18 patients with hepatitis C virus (HCV). Furthermore, 28 cases had diabetes, and139 cases were alcohol abuser. Overall, 4,956 MDR *M. tuberculosis* strains were isolated from TB patients. The pooled frequency of LNZ resistance among the clinical isolates of MDR *M. tuberculosis* was 4.2% (95%). Begg’s (*p* = 0.72) test showed no evidence of publication bias.

**Conclusion:** LNZ resistance among MDR M. tuberculosis isolates is increasing. On the other hand, long-term treatment of MDR-TB cases with LNZ alone is associated with several adverse effects. Thus, it is recommended that newer anti-TB drugs, including bedaquiline and delamanid, in combination with linezolid could increase its effectiveness and decrease toxicities. However, more studies should be done in this field.

## 1 Introduction

*Mycobacterium tuberculosis* (*M. tuberculosis*) is the leading cause of tuberculosis (TB), a disease with a major threat to the public health and millions of deaths worldwide (Azimi et al., 2017; Azimi et al., 2018). According to the 2020 global TB report by the World Health Organization (WHO), there were 10 million (range: 8.9–11.0 million) new TB cases and 1.2 million (range: 1.1–1.3 million) TB deaths among human immunodeficiency virus (HIV)-negative people in 2019 (WHO, 2020). The emergence of drug-resistant TB poses a danger to TB control throughout the world (Ahmed et al., 2013).

Isoniazid and rifampin are two effective and standard anti-TB drugs. *M. tuberculosis* strains resistant to these two antibiotics are defined as multidrug-resistant tuberculosis (MDR-TB) (Eker et al., 2008; Rey-Jurado et al., 2013; Guglielmetti et al., 2015). Based on the WHO estimates, virtually 3.3% of new TB cases and 17.7% of previously treated TB cases develop MDR-TB globally (WHO, 2020). Treatment of MDR-TB is performed using less efficient and more expensive second-line treatment drugs, with great adverse events. Moreover, unlike the treatment of drug-susceptible cases, that of MDR-TB needs a long period of time (Yang et al., 2018; Zong et al., 2018).

Linezolid (LNZ) is a member of the oxazolidinone class of antimicrobials that acquired the WHO license for clinical use. In 2016, the WHO recommended LNZ as the most important second-line drug for treating MDR-TB (Zong et al., 2018). Several *in vitro* and *in vivo* studies have revealed that LNZ has promising bacteriostatic activity against MDR-TB (Alcalá et al., 2003; Dietze et al., 2008; Tsona et al., 2010; Strydom et al., 2019). LNZ binds to domain V of 23S rRNA on the 50S ribosomal subunit, inhibits the formation of a 70S initiation complex, and prevents bacterial protein synthesis. It is presumed that mutation in two ribosomal proteins L3 and L4 and 23S rRNA is the main mechanism used by MDR-TB isolates, conferring resistance to LNZ (Zhang et al., 2014). In recent years, resistance to LNZ among MDR-TB cases has been reported in various countries (Richter et al., 2007; Pang et al., 2017). To this end, we performed a systematic review and meta-analysis to investigate the prevalence of LNZ resistance among MDR-TB isolates across the world.

## 2 Materials and methods

### 2.1 Search strategy

The present systematic and meta-analysis study was carried out based on the Preferred Reporting Items for Systematic Review and Meta-Analyses (PRISMA) guidelines. To evaluate the prevalence of LNZ resistance among MDR-TB isolates, we conducted a literature search in three main electronic databases, including PubMed/Medline, Web of Science, and Embase, from 1 January 2000 to 12 April 2021. We performed the searching process using the following keywords: “Mycobacteriaceae”, “mycobacteria”, “*mycobacterium*”, “*Mycobacterium tuberculosis*”, “tubercle *bacillus*”, “pulmonary tuberculosis”, “tuberculosis”, “Multidrug Resistance”, “Multi-Drug Resistance”, “Multiple drug resistance”, “MDR”, “multiresistance”, “linezolid”, “linezolid”, “oxazolidinone class antibiotic”, and “protein synthesis inhibitors”. The systematic search process was accomplished based on the medical subject heading (MeSH) terms alone or in combination with “AND” and/or “OR”. The search was limited to the original articles published in English and indicated the prevalence or incidence of LNZ resistance among MDR *M. tuberculosis* isolates. We also searched the bibliographies for any retrieved articles for additional references.

### 2.2 Inclusion criteria

The inclusion criteria included all the original papers presenting cross-sectional or cohort studies on the prevalence of LNZ resistance among MDR-TB. The selected studies were analyzed in terms of titles, abstracts, and full texts. The entire recorded studies included in our analysis based on the following criteria: original articles that provided sufficient data on LNZ resistance in MDR *M. tuberculosis* isolates, and those that used standard (conventional and molecular) methods. The conventional methods encompassed niacin accumulation, growth in Lowenstein-Jensen (LJ) media containing thiophene-carboxylic acid hydrazide (TCH), growth at 42 and 44°C, tolerance towards NaCl 5% and urease, pigment production in light and dark, catalase, arylsulfatase activity, Tween hydrolysis, tellurite reduction, and nitrate reduction, and molecular methods embraced PCR-RFLP and sequencing.

### 2.3 Exclusion criteria

The exclusion criteria were comprised of review article, modelling study, commentary, correspondence, duplicate articles for the same investigation, editorial, guideline, news, and studies without enough data on LNZ resistance in MDR *M. tuberculosis* isolates.

### 2.4 Data extraction and definitions

In the first step, we saved all initial records collected during database searching in a Word file, and primary screening was performed based on their topics and/or abstracts. In the next step, the full-texts of potentially eligible records were downloaded. Then the inclusion criteria and final eligibility for the downloaded full texts were evaluated by two trained researchers (TA and MH). The full texts of potentially eligible articles were read objectively and with details by these two researchers. Any disagreement between the two researchers was resolved by the decision of a third trained researcher (MJN). The extraction of necessary data was accomplished by two authors (SK and AA), and all obtained data were rechecked by other authors (TA, MH, and MJN). After extracting from each relevant article, data, including first author, date of investigation, year of publication, country/continent, diagnostic method, number of MDR *M. tuberculosis*, number and percentage of LNZ resistance, drug susceptibility testing, and source of isolates, were saved in Excel software (Microsoft, Redmond, WA, United States).

### 2.5 Quality assessment

We assessed all reviewed studies based on the quality assessment checklist, which was provided by the Joanna Briggs Institute (Joanna Briggs Institute, 2014).

### 2.6 Meta-analysis

Meta-analysis was performed using Comprehensive Meta-Analysis (Biostat V2.2) software. The amount of residual heterogeneity was evaluated by the t-squared statistic and the Q-statistic to test the heterogeneity between the inquiries. To assess any possible publication bias, Begg’s rank correlation Egger’s weighted regression methods was used in combination with a funnel plot. The probability level of *p* < 0.05 was considered as statistically significant publication bias.

## 3 Results

### 3.1 Study characteristics

**FIGURE 1:**
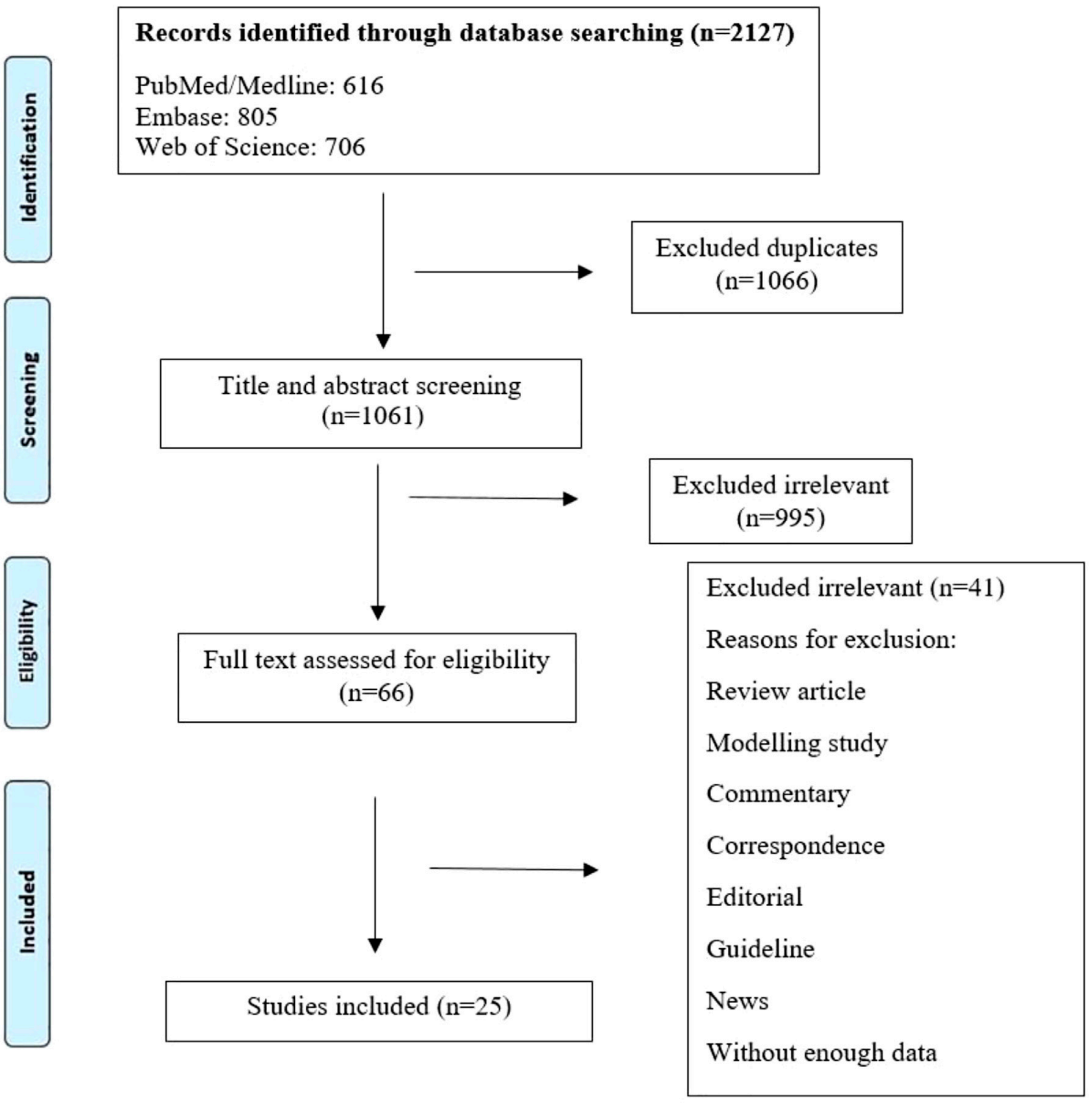
Flow diagram detailing review process and study selection.

Following the initial search of databases, a total of 2,127 articles were retrieved. After screenings the title/abstract and full texts, we selected 25 studies in the final evaluation (Figure 1). Table 1 depicts an overview of the main characteristics of the included studies. All the selected studies reported the prevalence of LNZ resistance among patients infected with MDR *M. tuberculosis* isolates, and mycobacteria were confirmed by microscopy, culture, and drug susceptibility tests. The location of studies covered all the continents except Africa. These studies were from 14 different countries, with the majority from the China (n = 5), Turkey (n = 4), United States (n = 2), Thailand (n = 2), France (n = 2), and Taiwan (n = 2). Other countries were comprised of Germany, Russia, Korea, Iran, India, Spain, India, and Italy, with one study. A total of 7,366 patients infected with MDR *M. tuberculosis* were enrolled in the current systematic review and meta-analysis. Of the study population, 98 patients were co-infected with HIV and 18 patients with hepatitis C virus (HCV). In addition, 139 cases were alcohol abuser, and 28 cases had diabetes.

**TABLE 1:** Characteristics of the included studies.

First author	Country	Published time	Number of patients	Number of MDR M. tuberculosis	Diagnosis method	Number of LNZ resistance (%)	DST method	Results of MIC (µg/ml)	Comorbidities
Eker Eker et al. (2008)	Germany	2008	177	177	Culture, Sputum smear	1 (0.6)	MGIT	NM	7 HIV-positive
Huang Huang et al. (2008)	Taiwan	2008	57	57	Culture	3 (5.3)	MIC	0.125–4	NM
Lai Lai et al. (2008)	Taiwan	2008	2,625	150	BACTEC MGIT 960 system, Culture	0 (0)	MIC	NM	NM
Coban Coban et al. (2009)	Turkey	2009	10	10	Culture	0 (0)	MICs	NM	NM
Limoncu Limoncu et al. (2011)	Turkey	2010	9	5	Culture, Sputum smear	0 (0)	agar dilution method, MIC	NM	NM
Pholwat Pholwat et al.(2011)	Thailand	2011	25	25	Culture	0 (0)	Bactec MGIT method	NM	NM
Bektore Bektöre et al, (2013)	Turkey	2012	81	81	BACTEC MGIT 960	0 (0)	BACTEC MGIT 960	NM	NM
Yang Yang et al. (2012)	China	2012	84	84	Culture	0 (0)	MIC	0.125–0.5	NM
Ahmed Ahmed et al. (2013)	Pakistan	2013	102	102	Culture, MGIT	6 (5.9)	MIC	1	NM
Rey-Jurado Rey-Jurado et al. (2013)	Spain	2013	9	9	Culture	2 (22.2)	MICs	1	NM
Chaiprasert Chaiprasert et al. (2014)	Thailand	2014	1,447	1,129	Culture	8 (0.7)	disc elution method	NM	NM
Zhang Zhang et al. (2014)	China	2014		107	PCR, Culture	17 (15.9)	MIC	32	NM
Cambau Cambau et al. (2015)	France	2015	139	139	MGIT	0 (0)	MGIT	NM	NM
Guglielmetti Guglielmetti et al. (2015)	France	2015	35	35	Culture, Sputum smear	0 (0)	MGIT	NM	18 HCV 7 Alcohol abuse
Simek Şimşek et al. (2015)	Turkey	2015	122	122	Culture	3 (2.4)	E-test method	NM	NM
Borisov Borisov et al. (2017)	Russia	2017	428	428	Sputum smear, Culture	4 (0.9)	MGIT	NM	91 HIV-positive 1 Pregnant 12 Thyroid disease 40 Heart disease 13 Pre-existing ECG abnormality 132 Alcoholism 75 Drug abuse 26 Diabetes
Cavanaugh Cavanaugh et al. (2017)	United States	2017		228	Culture	0 (0)	MIC	NM	NM
Yang Yang et al. (2018)	Korea	2018	420	171	PCR	1 (0.6)	MIC	1	NM
Zong Zong et al. (2018)	China	2018	120	120	Culture	10 (8.3)	MIC	16	NM
Gavali Gavali et al. (2019)	India	2019	468	468	Culture	14 (3)	MGIT tube	NM	NM
Riccardi Riccardi et al. (2019)	Italy	2019	134	134	Culture	2 (1.5)	MGIT	NM	NM
Kardan-Yamchi (Kardan-Yamchi et al. (2020)	Iran	2020	35	35	Culture	0 (0)	WGS	NM	NM
Tornheim Tornheim et al. (2020)	United States	2020		343	Smear, Xpert MTB/RIF, line probe assays and pyrosequencing	23 (6.7)	MGIT	1	2 Diabetes
Wang Wang et al. (2021)	China	2021	391	391	Culture	15 (3.8)	WGS	NM	NM
Zheng Zheng et al. (2021)	China	2021	88	88	Sputum smear	4 (4.5)	MIC	2	NM

AbbreviationsMDR: multidrug-resistant, MIC: minimum inhibitory concentration, DST: drug susceptibility testing, LNZ: linezolid, HIV: human immunodeficiency virus, MGIT: mycobacterial Growth Indicator Tube, HCV: hepatitis C virus, NM: not mentioned, PCR: polymerase chain reaction, ECG: electrocardiogram, MTB: mycobacterium tuberculosis, RIF: rifampin, WGS: whole genome sequencing.

### 3.2 The overall prevalence of LNZ resistance

**FIGURE 2:**
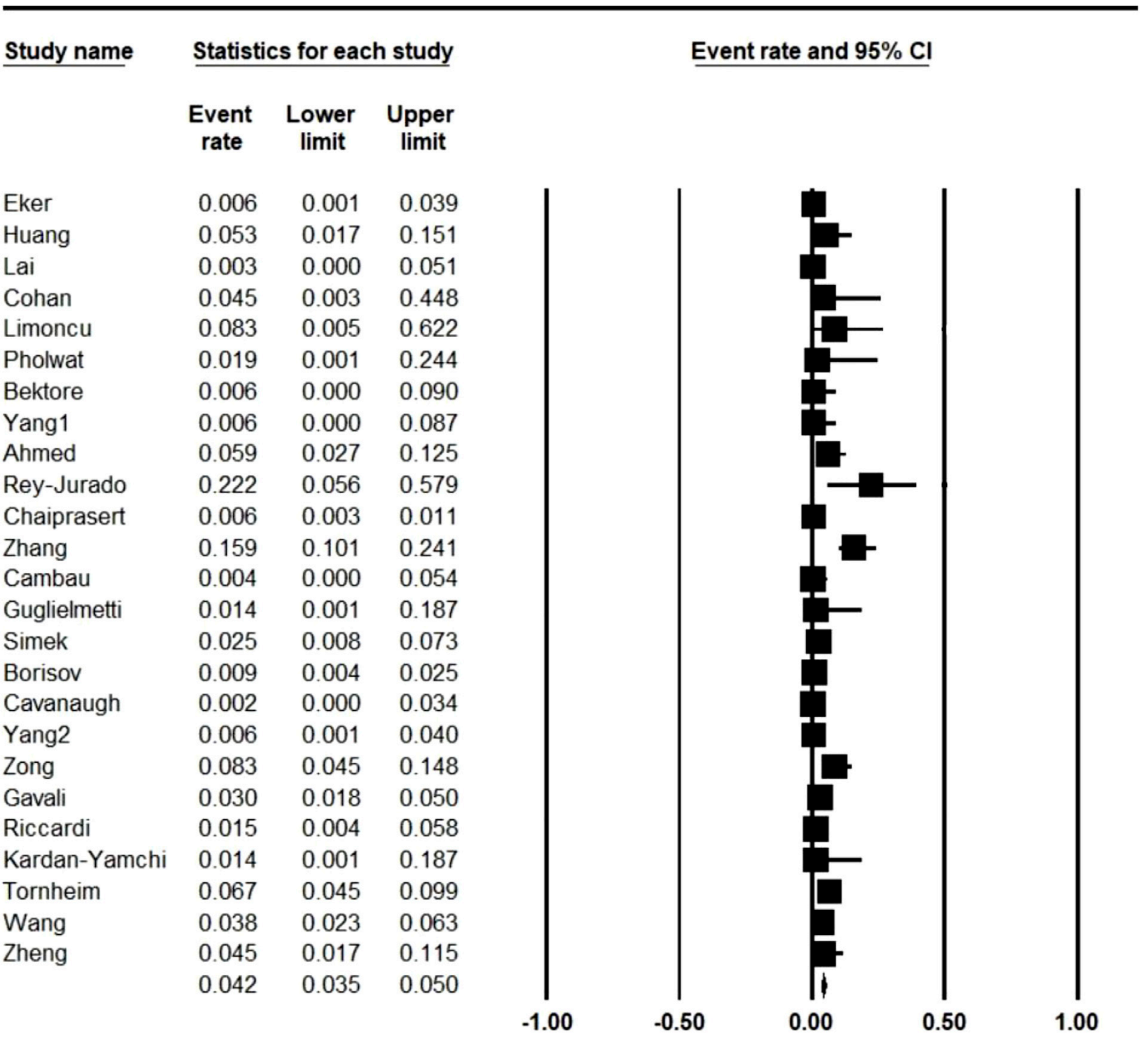
Pooled prevalence of linezolid resistance in multidrug-resistant *Mycobacterium tuberculosis* isolates. CI: confidence interval.

In total, 4,956 MDR *M. tuberculosis* strains were isolated from the patients with TB. Further analysis showed that 113 strains were LNZ resistant. As illustrated in Figure 2, the pooled frequency of LNZ resistance among the clinical isolates of MDR *M. tuberculosis* was 4.2% (95% CI: 3.5–5.0).

### 3.3 Prevalence of LNZ resistance in MDR M. tuberculosis by countries

As presented in Table 1 and based on studies carried out in different countries, Spain (22.2%; 95% CI: 5.6–57.9) and United States (0.2%; 95% CI: 0.0–3.4) had the highest and lowest rate of resistance to LNZ in MDR *M. tuberculosis* isolates, respectively. We calculated the resistance to LNZ in MDR *M. tuberculosis* isolates among countries with more than one study and per group of years (Table 2). Our analyses revealed that China with five studies (LNZ resistance: 5.8%) and France with two studies (LNZ resistance: 0%) had the highest and lowest rate of resistance to LNZ, respectively.

**TABLE 2:** Prevalence of linezolid resistance in MDR M. tuberculosis by countries.

Country	Number of study	Number of MDR *M. tuberculosis*	Number of LNZ resistance (%)
Taiwan	2	207	3 (1.4)
Turkey	4	218	3 (1.4)
Thailand	2	1,154	8 (0.7)
China	5	790	46 (5.8)
France	2	174	0 (0)
United States	2	571	23 (4)

AbbreviationsMDR: multidrug-resistant, LNZ: linezolid.

### 3.4 Time trend analysis of LNZ resistance in MDR *M. tuberculosis*

As time trend analysis of LNZ resistance in MDR *M. tuberculosis* is shown in Table 3, there was no specific pattern of LNZ resistance during 2008–2021. However, variation in LNZ resistance during the mentioned time period is considerable. Altogether, the rate of LNZ resistance during 2008–2012 was lower (0%) than that of other years. The highest rate of LNZ resistance was reported in 2013 (7.2%), followed by 2021 (6%).

**TABLE 3:** Time trend analysis of linezolid resistance in MDR M. tuberculosis

Group of years	Number of study	Number of MDR *M. tuberculosis*	Number of LNZ resistance (%)
2008	3	384	4 (1)
2009	1	10	0 (0)
2010	1	5	0 (0)
2011	1	25	0 (0)
2012	2	165	0 (0)
2013	2	111	8 (7.2)
2014	2	1,236	25 (2)
2015	3	296	3 (1)
2017	2	656	4 (0.6)
2018	2	291	11 (3.8)
2019	2	602	16 (2.6)
2020	2	378	23 (6)
2021	2	479	19 (4)

Abbreviations: MDR: multidrug-resistant, LNZ: linezolid.

### 3.5 Publication bias

**FIGURE 3:**
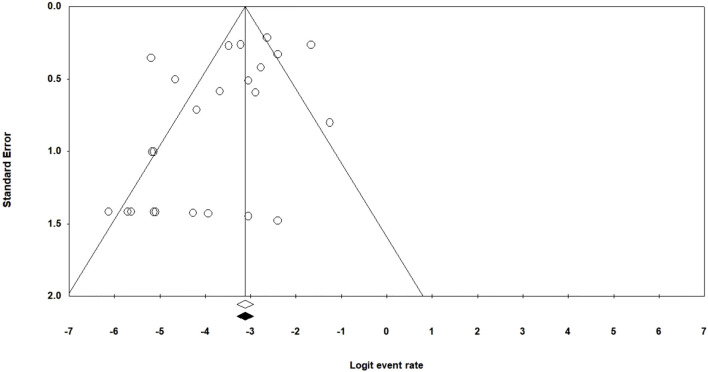
Funnel plot of the meta-analysis of Linezolid resistance in multidrug-resistant *Mycobacterium tuberculosis* isolates.

Figure 3 provides a funnel plot of standard error. From the data in this Figure and according to Begg’s (*p* = 0.72) test, it can be found that there was not evidence of publication bias.

## 4 Discussion

Globally, drug resistance among *M. tuberculosis* is increasing, and surveillance information reveals that MDR-TB has become an emerging and a major public health problem in many countries (Ahmed et al., 2013). Treatment of MDR-TB is exceedingly difficult and requires a prolonged time and multiple second-line drugs. Moreover, the lack of a standard regime for the treatment of MDR-TB infection can lead to the widespread person-to-person spread of drug-resistant organisms (Borisov et al., 2017). In the present systematic and meta-analysis study, the worldwide prevalence of LNZ resistance among MDR-TB isolates was investigated from 2000 to 2021. To the best of our knowledge, the current research is the first comprehensive systematic review on the prevalence of LNZ resistance among MDR-TB isolates in the world. The main goal of this study is to help the reduction of antibiotic resistance and effective control of infectious disease outbreaks.

The current meta-analysis study revealed that the overall estimate of LNZ resistance among clinical isolates of MDR-TB was 4.2%. Likewise, LNZ resistance rate among the clinical isolates of MDR-TB in 14 different countries showed that Spain (22.2%) and United States (0.2%) had the highest and lowest prevalence rate, respectively. Altogether, our analyses suggested that the LNZ resistance rate is 4.2%. In most cases, resistance to LNZ is related to four types of mutation, including T460C in rplC, G2576T, G2447T, and G2061T in 23 S rRNA (Zong et al., 2018). Moreover, the efflux pumps are another factor giving rise to low-level resistance to LNZ among extensively drug-resistant tuberculosis (XDR-TB) isolates (Zhang et al., 2014). In general, LNZ has bacteriostatic activity against *M. tuberculosis*, and with great penetration into infected tissues, such as lungs and cerebrospinal fluid, this drug can be selected as a promising antibiotic for treating MDR-TB and XDR-TB (Tornheim et al., 2020). However, it has been indicated that the long-term treatment of *M. tuberculosis* infections with LNZ alone is associated with several adverse effects, particularly painful peripheral neuropathy, optic neuritis, vision-threatening, myelosuppression, and drug interactions (Zong et al., 2018; Gavali et al., 2019). Many different published *in vivo* and *in vitro* studies have explored that LNZ in combination with other antimicrobials, e.g. delpazolid, rifampicin, isoniazid, fluoroquinolones, bedaquiline, amikacin, and clofazimine, has effective outcomes and high safety profiles and can improve treatment consequences (Limoncu et al., 2011; Rey-Jurado et al., 2013; Zong et al., 2018). It has been revealed that combination therapy could reduce the length of treatment of active tuberculosis. Furthermore, in asymptomatic patients, combination therapy could destroy the latent *M. tuberculosis* isolates (Limoncu et al., 2011). The current systematic and meta-analyses review was performed on 25 studies from 14 different countries. Therefore, the main limitation of our study was the lack of further studies from more different countries and regions of the world.

## 5 Conclusion

Our analyses uncovered that the LNZ resistance among MDR-TB is 4.2%, and this antimicrobial drug is used as an anti-TB drug in treatment regimes. However, considering several adverse events reported in studies, combination therapy could reduce LNZ toxicities associated with overdose concentrations. We believe that combination therapy, including LNZ plus rifampicin, LNZ plus isoniazid, linezolid-delpazolid, or LNZ with bedaquiline and fluoroquinolones or clofazimine, could have the highest synergistic result against MDR-TB and XDR-TB isolates compared to LNZ alone. However, additional comparative studies are needed to be conducted in different countries to recognize the possible profits of combination therapies in treating MDR-TB and XDR-TB isolates.

## Data Availability

The raw data supporting the conclusions of this article will be made available by the authors, without undue reservation.

## References

[B1] Ahmed I. Jabeen K. Inayat R. Hasan R. (2013). Susceptibility testing of extensively drug-resistant and pre-extensively drug-resistant *Mycobacterium tuberculosis* against levofloxacin, linezolid, and amoxicillin-clavulanate. Antimicrob. Agents Chemother. 57 (6), 2522–2525. 10.1128/AAC.02020-12 23507286 PMC3716178

[B2] Alcalá L. Ruiz-Serrano M. J. Turégano C. P-F. De Viedma D. G. Díaz-Infantes M. Marin-Arriaza M. (2003). *In vitro* activities of linezolid against clinical isolates of *Mycobacterium tuberculosis* that are susceptible or resistant to first-line antituberculous drugs. Antimicrob. Agents Chemother. 47 (1), 416–417. 10.1128/aac.47.1.416-417.2003 12499228 PMC148996

[B3] Azimi T. Nasiri M. J. Zamani S. Hashemi A. Goudarzi H. Fooladi A. A. I. (2018). High genetic diversity among *Mycobacterium tuberculosis* strains in Tehran, Iran. J. Clin. Tuberc. Other Mycobact. Dis. 11, 1–6. 10.1016/j.jctube.2018.01.001 31720383 PMC6830142

[B4] Azimi T. Shariati A. Fallah F. Imani Fooladi A. A. Hashemi A. Goudarzi H. (2017). *Mycobacterium tuberculosis* genotyping using MIRU-VNTR typing. J. Mazandaran Univ. Med. Sci. 27 (149), 40–48.

[B5] Bektöre B. Haznedaroglu T. Baylan O. Ozyurt M. Ozkutuk N. Satana D. (2013). Investigation of extensive drug resistance in multidrug resistance tuberculosis isolates. Mikrobiyol. Bul. 47 (1), 59–70. 10.5578/mb.4229 23390903

[B6] Borisov S. E. Dheda K. Enwerem M. Leyet R. R. D'Ambrosio L. Centis R. (2017). Effectiveness and safety of bedaquiline-containing regimens in the treatment of MDR-and XDR-TB: A multicentre study. Eur. Respir. J. 49 (5), 1700387. 10.1183/13993003.00387-2017 28529205

[B7] Cambau E. Viveiros M. Machado D. Raskine L. Ritter C. Tortoli E. (2015). Revisiting susceptibility testing in MDR-TB by a standardized quantitative phenotypic assessment in a European multicentre study. J. Antimicrob. Chemother. 70 (3), 686–696. 10.1093/jac/dku438 25587993

[B8] Cavanaugh J. S. Jou R. Wu M-H. Dalton T. Kurbatova E. Ershova J. (2017). Susceptibilities of MDR *Mycobacterium tuberculosis* isolates to unconventional drugs compared with their reported pharmacokinetic/pharmacodynamic parameters. J. Antimicrob. Chemother. 72 (6), 1678–1687. 10.1093/jac/dkx022 28333192 PMC5890777

[B9] Chaiprasert A. Srimuang S. Tingtoy N. Makhao N. Sirirudeeporn P. Tomnongdee N. (2014). Second-line drug susceptibilities of multidrug-resistant tuberculosis strains isolated in Thailand: An update. Int. J. Tuberc. Lung Dis. 18 (8), 961–963. 10.5588/ijtld.13.0197 25199012

[B10] Coban A. Y. Bilgin K. Uzun M. Durupinar B. (2009). Effect of linezolid in combination with isoniazid and rifampicin against multidrug resistant *Mycobacterium tuberculosis* clinical isolates. Mikrobiyol. Bul. 43 (2), 293–297. 19621615

[B11] Dietze R. Hadad D. J. McGee B. Molino L. P. D. Maciel E. L. N. Peloquin C. A. (2008). Early and extended early bactericidal activity of linezolid in pulmonary tuberculosis. Am. J. Respir. Crit. Care Med. 178 (11), 1180–1185. 10.1164/rccm.200806-892OC 18787216 PMC2588492

[B12] Eker B. Ortmann J. Migliori G. B. Sotgiu G. Muetterlein R. Centis R. (2008). Multidrug-and extensively drug-resistant tuberculosis, Germany. Emerg. Infect. Dis. 14 (11), 1700–1706. 10.3201/eid1411.080729 18976552 PMC2630755

[B13] Gavali D. Aring B. Mullan S. Nathametha A. A. Dave A. B. (2019). Evaluation of sensitivity and resistance of linezolid in pre extensively drug resistance tuberculosis and extensively drug resistance tuberculosis at a tertiary care hospital, jamnagar, Gujarat, India. J. Clin. Diagnostic Res. 13, 11. 10.7860/JCDR/2019/42544.13298

[B14] Guglielmetti L. Le Du D. Jachym M. Henry B. Martin D. Caumes E. (2015). Compassionate use of bedaquiline for the treatment of multidrug-resistant and extensively drug-resistant tuberculosis: Interim analysis of a French cohort. Clin. Infect. Dis. 60 (2), 188–194. 10.1093/cid/ciu786 25320286

[B15] Huang T-S. Liu Y-C. Sy C-L. Chen Y-S. Tu H-Z. Chen B-C. (2008). *In vitro* activities of linezolid against clinical isolates of *Mycobacterium tuberculosis* complex isolated in Taiwan over 10 years. Antimicrob. Agents Chemother. 52 (6), 2226–2227. 10.1128/AAC.00414-07 18391030 PMC2415800

[B16] Joanna Briggs Institute (2014). Joanna Briggs Institute reviewers’ manual: 2014 edition. Australia: The Joanna Briggs Institute.

[B17] Kardan-Yamchi J. Kazemian H. Battaglia S. Abtahi H. Foroushani A. R. Hamzelou G. (2020). Whole genome sequencing results associated with minimum inhibitory concentrations of 14 anti-tuberculosis drugs among rifampicin-resistant isolates of mycobacterium tuberculosis from Iran. J. Clin. Med. 9 (2), 465. 10.3390/jcm9020465 PMC7073636 32046149

[B18] Limoncu M. H. Ermertcan Ş. Erac B. Taşli H. (2011). An investigation of the antimicrobial impact of drug combinations against *Mycobacterium tuberculosis* strains. Turkish J. Med. Sci. 41 (4), 719–724. 10.3906/sag-1007-934

[B19] Lai C-C. Tan C-K. Huang Y-T. Chou C-H. Hung C-C. Yang P-C. (2008). Extensively drug-resistant *Mycobacterium tuberculosis* during a trend of decreasing drug resistance from 2000 through 2006 at a medical center in Taiwan. Clin. Infect. Dis. 47 (7), e57–e63. 10.1086/591702 18715157

[B20] Pang Y. Zong Z. Huo F. Jing W. Ma Y. Dong L. (2017). *In vitro* drug susceptibility of bedaquiline, delamanid, linezolid, clofazimine, moxifloxacin, and gatifloxacin against extensively drug-resistant tuberculosis in Beijing, China. Antimicrob. Agents Chemother. 61 (10), e00900–17. 10.1128/AAC.00900-17 28739779 PMC5610515

[B21] Pholwat S. Heysell S. Stroup S. Foongladda S. Houpt E. (2011). Rapid first-and second-line drug susceptibility assay for *Mycobacterium tuberculosis* isolates by use of quantitative PCR. J. Clin. Microbiol. 49 (1), 69–75. 10.1128/JCM.01500-10 21084506 PMC3020429

[B22] Rey-Jurado E. Tudó G. de la Bellacasa J. P. Espasa M. Gonzalez-Martín J. (2013). *In vitro* effect of three-drug combinations of antituberculous agents against multidrug-resistant *Mycobacterium tuberculosis* isolates. Int. J. Antimicrob. Agents 41 (3), 278–280. 10.1016/j.ijantimicag.2012.11.011 23312604

[B23] Riccardi N. Alagna R. Saderi L. Ferrarese M. Castellotti P. Mazzola E. (2019). Towards tailored regimens in the treatment of drug-resistant tuberculosis: A retrospective study in two Italian reference centres. BMC Infect. Dis. 19 (1), 564. 10.1186/s12879-019-4211-0 31253115 PMC6599241

[B24] Richter E. Rüsch-Gerdes S. Hillemann D. (2007). First linezolid-resistant clinical isolates of *Mycobacterium tuberculosis* . Antimicrob. Agents Chemother. 51 (4), 1534–1536. 10.1128/AAC.01113-06 17242139 PMC1855508

[B25] Şimşek H. Tarhan G. Cesur S. (2015). Evaluation of second-line antituberculosis drug susceptibilities of multidrug-resistant mycobacterium tuberculosis complex isolates by E-test method. Mikrobiyol. Bul. 49 (1), 47–55. 10.5578/mb.8602 25706730

[B26] Strydom N. Gupta S. V. Fox W. S. Via L. E. Bang H. Lee M. (2019). Tuberculosis drugs’ distribution and emergence of resistance in patient’s lung lesions: A mechanistic model and tool for regimen and dose optimization. PLoS Med. 16 (4), e1002773. 10.1371/journal.pmed.1002773 30939136 PMC6445413

[B27] Tornheim J. A. Intini E. Gupta A. Udwadia Z. F. (2020). Clinical features associated with linezolid resistance among multidrug resistant tuberculosis patients at a tertiary care hospital in Mumbai, India. J. Clin. Tuberc. Other Mycobact. Dis. 20, 100175. 10.1016/j.jctube.2020.100175 32775702 PMC7398971

[B28] Tsona A. Metallidis S. Foroglou N. Selviaridis P. Chrysanthidis T. Lazaraki G. (2010). Linezolid penetration into cerebrospinal fluid and brain tissue. J. Chemother. 22 (1), 17–19. 10.1179/joc.2010.22.1.17 20227987

[B29] Wang G. Jiang G. Jing W. Zong Z. Yu X. Chen S. (2021). Prevalence and molecular characterizations of seven additional drug resistance among multidrug-resistant tuberculosis in China: A subsequent study of a national survey. J. Infect. 82 (3), 371–377. 10.1016/j.jinf.2021.02.004 33556430

[B30] WHO (2020). Global tuberculosis report 2020: Executive summary. Report.

[B31] Yang C. Lei H. Wang D. Meng X. He J. Tong A. (2012). *In vitro* activity of linezolid against clinical isolates of *Mycobacterium tuberculosis*, including multidrug-resistant and extensively drug-resistant strains from Beijing, China. Jpn. J. Infect. Dis. 65 (3), 240–242. 10.7883/yoken.65.240 22627306

[B32] Yang J. S. Kim K. J. Choi H. Lee S. H. (2018). Delamanid, bedaquiline, and linezolid minimum inhibitory concentration distributions and resistance-related gene mutations in multidrug-resistant and extensively drug-resistant tuberculosis in Korea. Ann. Lab. Med. 38 (6), 563–568. 10.3343/alm.2018.38.6.563 30027700 PMC6056398

[B33] Zhang Z. Pang Y. Wang Y. Liu C. Zhao Y. (2014). Beijing genotype of *Mycobacterium tuberculosis* is significantly associated with linezolid resistance in multidrug-resistant and extensively drug-resistant tuberculosis in China. Int. J. Antimicrob. Agents 43 (3), 231–235. 10.1016/j.ijantimicag.2013.12.007 24439458

[B34] Zheng H. He W. Jiao W. Xia H. Sun L. Wang S. (2021). Molecular characterization of multidrug-resistant tuberculosis against levofloxacin, moxifloxacin, bedaquiline, linezolid, clofazimine, and delamanid in southwest of China. BMC Infect. Dis. 21 (1), 330. 10.1186/s12879-021-06024-8 33832459 PMC8028109

[B35] Zong Z. Jing W. Shi J. Wen S. Zhang T. Huo F. (2018). Comparison of *in vitro* activity and MIC distributions between the novel oxazolidinone delpazolid and linezolid against multidrug-resistant and extensively drug-resistant *Mycobacterium tuberculosis* in China. Antimicrob. Agents Chemother. 62 (8), e00165–18. 10.1128/AAC.00165-18 29844043 PMC6105784

